# The new earthquake locations and focal mechanisms catalogues for the western Ionian Sea, Italy

**DOI:** 10.1038/s41597-026-06979-w

**Published:** 2026-03-05

**Authors:** Tiziana Sgroi, Graziella Barberi, Alessandro Marchetti, Luca Gasperini, Alina Polonia

**Affiliations:** 1 Istituto Nazionale di Geofisica e Vulcanologia, Sezione Roma 2, Rome, Italy; 2 Istituto Nazionale di Geofisica e Vulcanologia (INGV), Osservatorio Etneo, Catania, Italy; 3 Istituto Nazionale di Geofisica e Vulcanologia, Osservatorio Nazionale Terremoti, Rome, Italy; 4 Istituto di Scienze Marine (ISMAR), CNR, Bologna, Italy

**Keywords:** Seismology, Tectonics

## Abstract

The Ionian Sea hosts the last remnant of Tethyan oceanic lithosphere subducting beneath Calabria, where active deformation generates significant seismicity, including earthquakes exceeding magnitude 7. The main tectonic structures - the Ionian Fault system, the Alfeo–Etna Fault system, and the Malta Escarpment - accommodate African–Eurasian plate convergence and margin segmentation under a transtensional regime. Here we present two new datasets of 3D earthquake locations and focal mechanism solutions to constrain fault kinematics in the Ionian region. The first dataset includes 5,240 relocated small-magnitude earthquakes (0.7 ≤ M_L_ ≤ 4.7) recorded between 1990 and 2019. Hypocentral locations were determined integrating travel-times data from land-based and seafloor seismic stations to improve spatial resolution. The second dataset comprises 421 new focal mechanism solutions (1.5 ≤ M_L_ ≤ 4.7) for earthquakes occurring in the Ionian Sea and adjacent areas.

## Background & Summary

The densely populated Western Ionian Sea coast is highly vulnerable to natural hazards, including earthquakes (sometimes tsunamigenic), landslides, and volcanic eruptions, the latter being linked to the nearly continuous activity of Mount Etna.

Historically and in recent times, some of the strongest earthquakes in the Mediterranean region have occurred in this area, including the largest events ever recorded in Italy: the 1169, Mw 6.6; 1693, Mw 7.4; 1908, Mw 7.1; 1990, Mw 5.7^1,2^.

Despite extensive research and the wealth of data collected during marine geological and geophysical surveys^3–5^ the accurate locations of offshore seismogenic sources and the causative faults of large historical earthquakes remain debated. A key challenge arises from the limitation of the seismic network in accurately locating moderate-to-low magnitude offshore earthquakes. Events occurring at sea are particularly prone to mislocation or non-detection due to inadequate azimuthal coverage, as land-based seismic stations alone cannot record these events with adequate accuracy^6,7^. These earthquakes are often affected by insufficient azimuthal coverage, leading to misdetection or non-detection of small-to-moderate magnitude events and significant challenges in determining their locations using only data recorded from land-based seismic stations^7^.

To overcome these limitations, seafloor observatories and Ocean Bottom Seismometers (OBS), integrated with data from land-based seismic networks, have been deployed in various regions worldwide to improve the offshore earthquake detection and locations^8–10^. In recent years, several seismological experiments have been conducted in the western Ionian Sea to expand the seismic network towards the offshore by deploying seafloor observatories and Ocean Bottom Seismometers and Hydrophones (OBS/H). These included the deployment of the NEMO-SN1 seafloor observatory (the operational nodes of the research infrastructure EMSO – European Multidisciplinary Seafloor and Water-Column Observatory, www.emso.eu), which operated in an autonomous acoustic-linked mode from 2002 to 2003^6,11^ and later in cable mode from 2012 to 2013^12,13^. This observatory facilitated the synchronous recording of time-series data for multidisciplinary studies and provided valuable insights into oceanic regions, particularly regarding volcanic and tectonic structures^7,13,14^.

More recently, the SEISMOFAULTS project (www.seismofaults.it) represented the first experiment specifically designed to investigate the active tectonic structures of the Calabrian Arc subduction complex (offshore eastern Sicily and southern Calabria) through the deployment of an OBS network comprising seven seafloor stations^15^, that recorded seismological data from May 2017 to May 2018^16^.

In this study, we present two datasets comprising 3D earthquake locations and focal mechanism solutions. The relocated 3D seismicity spanning 1990 to 2019, combined with the spatial distribution and computed focal mechanisms, can enhance our understanding of the sub-seafloor tectonic framework of the western Ionian Sea. These datasets offer new constraints on active deformation and regional tectonic evolution and are intended to support further geophysical and seismotectonic investigations.

## Methods

### The new earthquakes catalogue

**Fig. 1:**
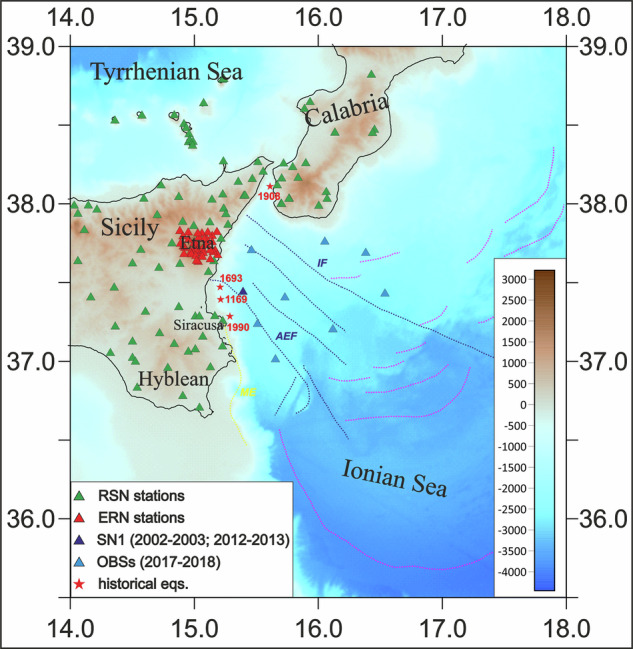
Shaded-relief morphological map of the Western Ionian Sea and surroundings from Ryan *et al*.^29^ with land and seafloor seismic stations represented by triangles (green: RSN; red: ERN; dark blue: NEMO-SN1 seafloor observatory and light blue: the SEISMOFAULTS’ OBSs; the deployment period of seafloor stations is indicated in the legend). The epicentres of the four among the highest magnitude earthquakes ever recorded in southern Italy^1,2^ (1169, Mw 6.6; 1693, Mw 7.4; 1908, Mw 7.1; 1990, Mw 5.7) are indicated with red stars and years. ME, AEF and IF indicate the main tectonic structures: the Malta escarpment (in yellow), and the Alfeo-Etna Fault and the Ionian Fault systems (in dark blue), respectively, while active thrust faults are depicted in magenta.

Our dataset consists of more than 5,000 crustal and subcrustal earthquakes that occurred in the western Ionian Sea, in correspondence of the eastern Sicily and southern Calabria coasts, between 1990–2019. We selected earthquakes within the rectangular area between 14.5°−18.0° longitude and 35.5°−38.5° latitude, collecting basic information (travel-times and first polarities) from available catalogues (https://bsi.ingv.it/it/archivio-dati; http://terremoti.ingv.it/; https://www.ct.ingv.it/index.php/monitoraggio-e-sorveglianza/banche-dati-terremoti/terremoti^17,18^) and from published studies^7,14,16,19^. Earthquakes were selected from two catalogues: the first containing data recorded by land stations of the “*Rete Sismica Nazionale”* (RSN)^20^, and the second from the Etna Regional Network (ERN), both managed by the Istituto Nazionale di Geofisica e Vulcanologia (INGV Seismological Data Centre and Osservatorio Etneo) (Fig. 1). As the two catalogues suffer from duplications, incompleteness, and errors in both geographic locations and focal depths, the first step consisted in eliminating the duplicates and merging the travel-times in case of common events.

The handpicked travel-times of NEMO-SN1 seafloor observatory^7,14^ and the SEISMOFAULTS OBS network data^16,19^ were integrated into the catalogue compiled using the land stations, resulting in a more extended dataset with a larger number of travel-times for earthquake location.

Moreover, earthquakes in the two seismic catalogues from RSN and ERN are located using 1D velocity models: the RSN uses a 1D velocity model^21^ valid for the entire national territory while the ERN uses a 1D velocity model^22^ at a local scale.

All earthquakes were relocated using the new 3D velocity model computed for the Ionian Sea^7^ and the tomoDDPS algorithm^23^. This software uses a combination of both absolute and differential P- and S-wave arrival times to improve the relative locations of closely spaced events. Arrival-time uncertainties, derived from picking quality, were used as weights in the relocation, and differential times reduce the effect of unmodeled velocity heterogeneity outside clusters. Hypocentral uncertainties are derived from the posterior covariance matrix provided by tomoDDPS, so that for closely spaced earthquakes, travel-times errors due to inaccurate velocity models outside the cluster are effectively cancelled. For event locations with tomoDDPS, we used: maximum station distance of 500 km, maximum neighboring distance of 10 km, maximum of 20 neighboring events, and a minimum of 6 arrival times per event. Arrival time uncertainties are considered in the computation of the probability for each hypocenter, while the final hypocentral uncertainties are derived from the covariance matrix calculated by the software, which accounts for network geometry and pick quality. All other parameters were kept at the program’s default values.

The tomographic model, used to relocate the earthquakes and computed on P-wave ray tracing^7^, shows a Moho depth of about 20 km in the central part of the Ionian basin, while depths up to 30 km have been computed in the coastal areas of eastern Sicily and southern Calabria. The crustal structure of the area is well resolved from the tomographic model down to about 40 km depth, whereas below 40 km and in the outermost sectors, the resolution and accuracy are limited by the event-station geometry and the poor coverage of seismic stations. This 3D velocity model is more suitable for crustal seismicity locations in the offshore relative to the previous velocity models, underlined by the seismicity distribution aligning on the active tectonic structures^7^.

As accurate depth is crucial for illuminating tectonic structures, particularly offshore where direct observations are lacking, we tested their reliability comparing the hypocenters of the entire dataset with seafloor elevation using the EMODnet Digital Bathymetry (DTM 2020; https://emodnet.ec.europa.eu/en/bathymetry), which has a resolution of 0.0625 Arc second, taking into account depth uncertainties derived from the 3D locations. Only four events out of 5,244 were excluded from the final catalogue because hypocenters fell within the water column, even though the differences between hypocentres and bathymetric elevations were in the order of a few tens of meters.

Since different magnitude scales have been adopted in seismological bulletins in the past, we standardized our values using the regression equation proposed in^14^, converting all magnitudes from M_d_ to M_L_. Additionally, as the tomoDDPS algorithm does not calculate the azimuthal GAP of seismic events, we determined this parameter using the VELEST software^24^. This algorithm was designed to jointly invert earthquake hypocenters and a one-dimensional velocity model using P- and S-wave arrival times. Here, we used the VELEST solely to compute the azimuthal GAP, with earthquake locations fixed at hypocenters previously determined by tomoDDPS. Default control settings were used, including a maximum station distance of 500 km. Hypocentral uncertainties were not modified, as they are not recalculated when locations are fixed.

**Fig. 2:**
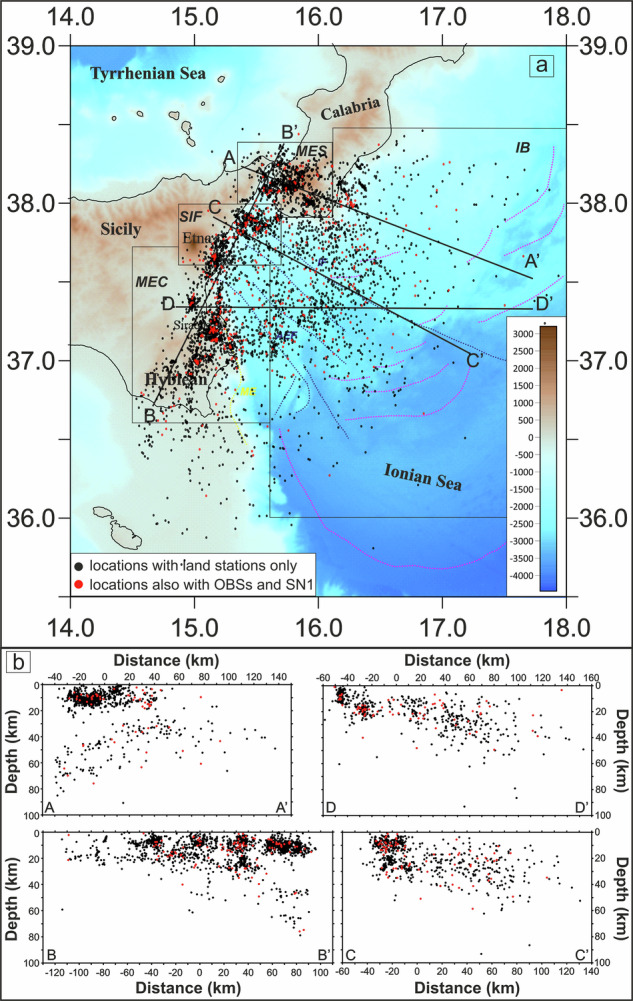
(**a**) Seismicity distribution in the Western Ionian Sea and surroundings according to the new catalogue published in this work, together with the main blocks (IB, MES, SIF, and MEC) identified on the basis of earthquake distributions (black and red circles). Earthquakes located using only arrival times from land stations are shown in black, while those located using arrival times from both marine (OBSs and NEMO-SN1) and land stations are shown in red. ME, AEF and IF indicate the main tectonic structures: the Malta escarpment (in yellow), and the Alfeo-Etna Fault and the Ionian Fault systems (in blue), while active thrust faults are depicted in magenta. (**b**) Cross-sectional view of the relocated seismicity. Events are projected along the four traces shown in (**a**) with black lines, using a swath width of ±10 km. Shaded-relief morphological map of the Western Ionian Sea and surrounding areas from Ryan *et al*.^29^.

The final catalogue (Fig. 2a) consists of 5,240 local earthquakes (0.7 ≤ *M*_*L*_ ≤ 4.7). Among these, 498 earthquakes (about the 10% of the dataset) were located using NEMO-SN1 and OBSs travel-times (red circles in Fig. 2a). These new locations enabled the creation of a fully comprehensive set of P- and S-wave arrivals, providing a more detailed characterization of the main tectonic structures (the Malta Escarpment, the Alfeo-Etna Fault, and Ionian Fault systems) compared with previous studies.

The earthquakes depth distribution is shown in sections of Fig. 2b, where a progressive deepening from the eastern coast of Sicily towards the Ionian Basin is observed and, in the outermost areas, earthquake depths exceed 100 kilometres.

### The new focal mechanism catalogue

Earthquake focal mechanisms are essential in seismotectonic studies and play a crucial role in understanding the relationship between earthquakes and their causative faults within their geodynamic framework. We computed focal mechanism solutions for events occurring in the western Ionian Sea, during the 1990–2019 period, using the FPFIT standard procedure^25^, which determines fault-plane solutions by minimizing the misfit between observed and theoretical P-wave first-motion polarities. We followed the standard FPFIT procedure as originally described, using take-off angles calculated from the 3D velocity model obtained from tomoDDPS relocations. Depth uncertainties for the selected events are generally smaller than 2 km for more than 95% of the dataset. Such uncertainties result in limited variations of the computed takeoff angles, typically within a few degrees, which are unlikely to significantly affect first-motion polarity assignments and the resulting focal mechanism solutions. Uncertainties in strike, dip, and rake were estimated by exploring the range of acceptable solutions within the FPFIT misfit threshold.

For this analysis, we selected earthquakes with a minimum of eight clear polarities distributed homogeneously over the focal sphere; approximately 70% of the events have ten or more polarities. Homogeneous coverage was quantified using FPFIT’s Station Distribution Ratio (STDR), which in our dataset ranges from 0.26 to 0.94. Only events with sufficient azimuthal and incidence angle coverage were included to ensure reliable solutions.

From a total of 421 new focal mechanisms (Dataset 2; 1.5 ≤ M_L_ ≤ 4.7), 92 (approximately 22% of the total) were computed using polarities detected on records from seafloor stations, resulting in an increase in the number of available focal mechanisms for earthquake kinematics in the Ionian Sea. We defined the quality factor (*Q*) of focal solutions based on the degree of polarity misfit, the number of discrepant stations, the Station Distribution Ratio (STDR), and the uncertainties in the strike, dip, and rake. In our dataset, the best-quality solutions (Q = 2) include 162 events with mean uncertainties ranging from 0° to 31° and STDR values between 0.35 and 0.87. Intermediate-quality solutions (Q = 1) comprise 257 events with mean uncertainties from 0° to 44° and STDR values from 0.26 to 0.94, while low-quality solutions (Q = 0) include 2 events with mean uncertainties of 8° and 37° and STDR values of 0.26 and 0.91. Strike uncertainties for all solutions range from 0° to 43°, dip from 0° to 63°, and rake from 0° to 80°, with the mean uncertainty across all parameters spanning 0° to 44°. These values reflect the actual distribution of uncertainties and STDR in our dataset.

Using the Frohlich (1992) classification scheme^26^, which is based on the plunge of P- and T-axes, we identified five kinematic categories: thrust, thrust-strike, strike-slip, normal, and normal-strike, along with a type of mechanism exhibiting horizontal/vertical axes. While a few events show thrust (31 earthquakes), thrust-strike (29), and horizontal-vertical (36) kinematics, the predominant kinematics are normal (97 earthquakes), normal/oblique (100), and strike-slip (128), which are observed at all depths.

**Fig. 3:**
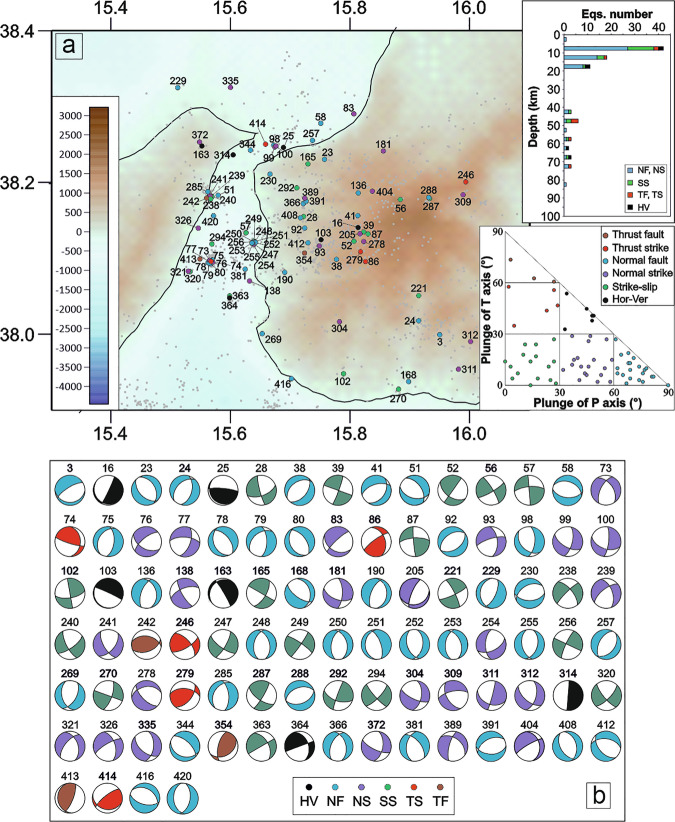
Seismicity close-up over the Messina Straits (MES) area. (**a**) Location map of relocated earthquakes and the new 94 focal mechanisms computed in the MES area; histogram of the number of focal mechanisms for each group and the corresponding kinematics versus depths (upper right) and distribution of the focal mechanisms in the kinematic classification based on the plunge of P- and T-axes (^26^; bottom right). The underlying shaded-relief morphological map of the Western Ionian Sea and surroundings is from Ryan *et al*.^29^. (**b**) The 94 FPSs computed for the MES.

**Fig. 4:**
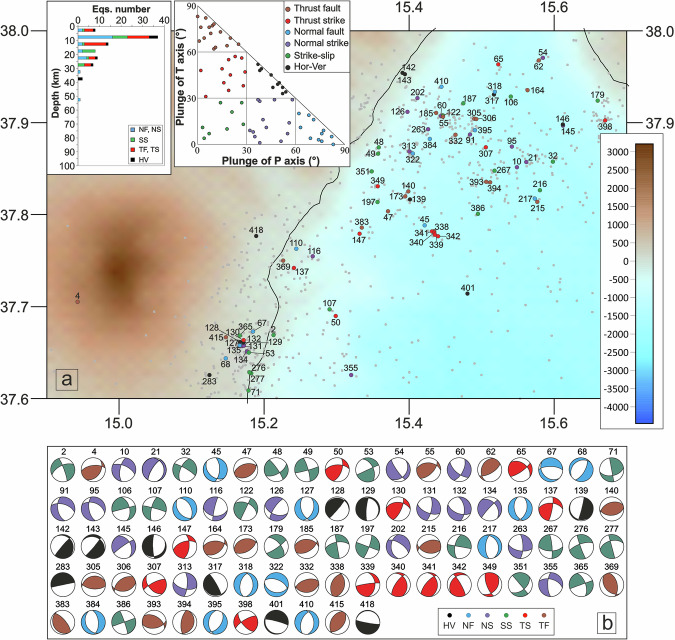
Seismicity occurred along the southern Ionian Fault (SIF). (**a**) Location map of relocated earthquakes and the new 87 focal mechanisms computed in the SIF area; histogram of the number of focal mechanisms for each group and the corresponding kinematics versus depths (upper left) and distribution of the focal mechanisms in the kinematic classification based on the plunge of P- and T-axes (^26^; upper right sketch). The underlying shaded-relief morphological map of the Western Ionian Sea and surroundings is from Ryan *et al*.^29^. (**b**) The 87 FPSs computed for the SIF.

**Fig. 5:**
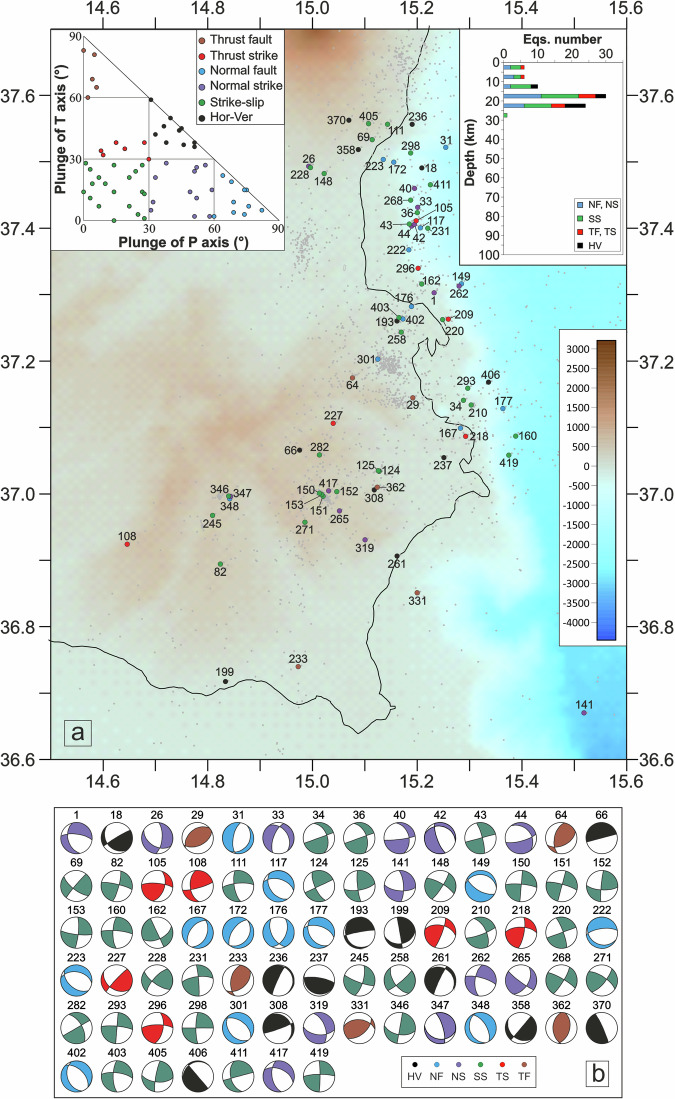
Seismicity in the Hyblean Plateau and along the Malta Escarpment (MEC). (**a**) Location map of relocated earthquakes and the new 77 focal mechanisms computed in the MEC area; histogram of the number of focal mechanisms for each group and the corresponding kinematics versus depths (upper right) and distribution of the focal mechanisms in the kinematic classification based on the plunge of P- and T-axes (^26^; upper left). The underlying shaded-relief morphological map of the Western Ionian Sea and surroundings is from Ryan *et al*.^29^ (**b**) The 77 FPSs computed for the MEC.

**Fig. 6:**
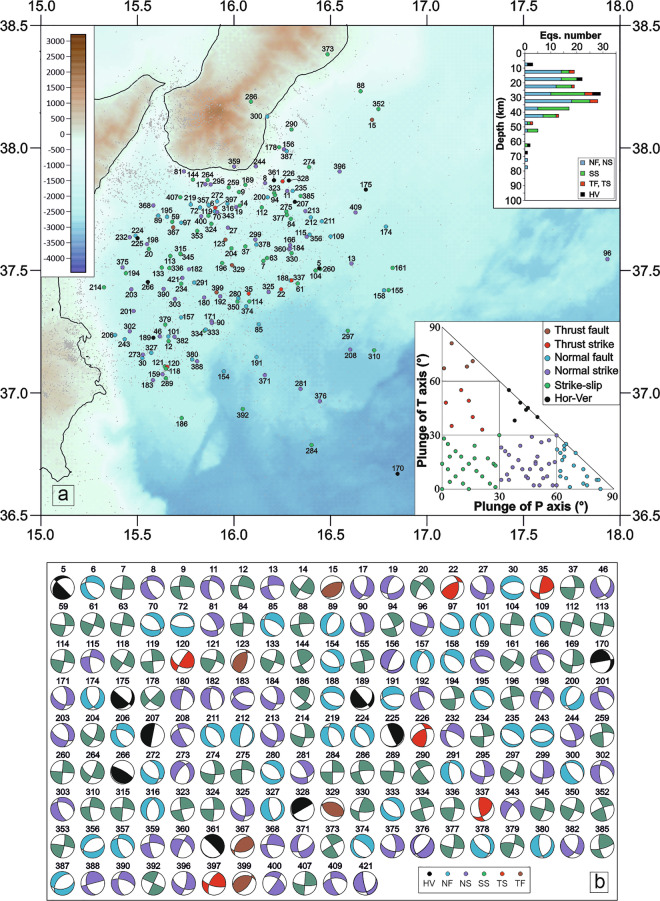
Seismicity in the Ionian Basin (IB). (**a**) Location map of relocated earthquakes and the new 163 focal mechanisms computed in the IB area; histogram of the number of focal mechanisms for each group and the corresponding kinematics versus depths (upper right) and distribution of the focal mechanisms in the kinematic classification based on the plunge of P- and T-axes (^26^; bottom right). The underlying shaded-relief morphological map of the Western Ionian Sea and surroundings is from Ryan *et al*.^29^. (**b**) The 163 FPSs computed for the IB.

To facilitate the visualization and comparison of focal mechanism distributions across structurally distinct domains we show the distribution of focal mechanisms into four distinct blocks (Figs. 3–6). Boundaries were established by testing alternative configurations to reduce spatial overlap of seismicity between adjacent domains, resulting in the sectorization shown in Fig. 2a. From north to south, the defined blocks are: 1) the Messina Straits (MES); 2) the sector to the south of the Ionian Fault (SIF); 3) the area surrounding the Malta escarpment (MEC); 4) the Ionian basin (IB).

The MES block (Figs. 2 and 3) corresponds to the Messina Strait area. This block includes 94 events (1.6 ≤ M_L_ ≤ 4.5) with predominantly normal (36 focal mechanisms), normal/oblique (24), and strike-slip (20) kinematics. Only 8 focal mechanisms exhibit compressive and/or thrust/oblique kinematics, while 6 events are characterized by horizontal/vertical kinematics.

The SIF block (Figs. 2 and 4), located south of the Ionian Fault near the eastern coast of Sicily, shows a similar spatial extent and a comparable number of events as the MES block, with 87 earthquakes (1.5 ≤ M_L_ ≤ 4.3). This block includes 12 normal mechanisms, 16 normal/oblique, and 19 strike-slip mechanisms, along with 30 focal mechanisms characterized by compressive kinematics (thrust and thrust/strike). Additionally, 10 events exhibit horizontal/vertical kinematics.

The MEC block primarily corresponds to the Malta Escarpment (Figs. 2 and 5), where 77 new focal mechanisms solutions were computed. The earthquakes (1.6 ≤ M_L_ ≤ 4.7) predominantly exhibit normal (12 events), normal/oblique (12 events), and strike-slip (31 events) kinematics. Horizontal/vertical mechanisms (11 events) and compressive mechanisms (5 thrust faults and 6 thrust-strike) are also observed.

The IB block (Figs. 2 and 6) includes 163 focal mechanisms for earthquakes (1.6 ≤ M_L_ ≤ 4.5), with prevalent normal and normal/oblique kinematics (37 normal faults, 48 normal/oblique faults) and strike-slip mechanisms (58 events); few compressive mechanisms (5 thrust faults and 6 thrust/strike) and horizontal/vertical mechanisms (9 events) are also computed.

## Data Record

The earthquake catalogue presented in this study has been uploaded in the INGV repository under the title **WISeLoc_1990–2019** (**W**estern **I**onian **S**ea **e**arthquake **Loc**ations - 1990–2019) by Barberi *et al*.^27^. It is a Microsoft Excel worksheet consisting in 5,240 rows organized into 15 columns, each row describing a single earthquake event, while each column contains the earthquake parameters. Columns labels in the Microsoft Excel sheet are the following:

- A: ID (identification number) - Earthquakes were arranged in this file from the older to the most recent.
- B: DATE (year, month, day) - date type variables indicating the date of each earthquake.
- C: O.T. (ORIGIN TIME) - the origin time of each earthquake is indicated in terms of hour, minutes, seconds and cents.
- D: LAT; E: LONG - indicate the location (latitude and longitude in degrees) of each event (four decimal number).
- F: DEPTH - indicates the depth in kilometres of each individual earthquake (two decimal numbers).
- G: M_L_ - magnitudes (local magnitude; one decimal number) for the included earthquakes.
- H: GAP –the azimuthal GAP in degrees indicates the coverage of the network with respect to the earthquake, and is an indicator of the individual location quality.
- I: RMS - Root Main Square representing the errors in seconds of the origin time for each earthquake.
- J: NO – total number of P- and S-phases for each event.
- K: N P-Phases; L: N S-Phases - number of P- and S-phases used to locate the single earthquake.
- M: ERR-X; N: ERR-Y; O: ERR-Z - horizontal and vertical errors in kilometres of the location for each individual earthquake.
  The second Microsoft Excel worksheet uploaded in the INGV repository under the title **WISeFM_1990–2019** (**W**estern **I**onian **S**ea **e**arthquakes **F**ocal **M**echanisms - 1990–2019)^28^ includes the final compiled focal mechanism solutions mentioned in the manuscript. This file consists of sheets composed by 20 columns and 421 rows, each row describing a single focal mechanism solution for a given earthquake, while each column the parameters for the event. The column’s titles are the following:
- A: ID (identification number) - earthquakes were arranged in this file from the older to the most recent.
- B: DATE (year, month, day) - date type variables indicating the date of each earthquake.
- C: O.T. (ORIGIN TIME) - the origin time of each earthquake is indicated in terms of hour, minutes, seconds and hundredths.
- D: LAT; E: LONG - indicate the location (latitude and longitude in degrees) of each event (four decimal numbers).
- F: DEPTH - indicates the depth in kilometres of each individual earthquake (two decimal numbers).
- G: M_L_ - magnitudes (local magnitude; one decimal number) for the included earthquakes.
- H: N Polar - number of P-wave polarities used to compute the focal mechanisms.
- I: STRK; J: DIP; K: RAKE – these columns represent one of the two nodal planes of the focal mechanism solution for each earthquake; each column contains the corresponding strike, dip, and rake angles.
- L: STRK; M: DIP; N: RAKE – these columns represent the auxiliary nodal planes for the focal mechanism solution for each earthquake; each column contains a number for each mentioned individual parameter (strike, dip, and rake angles).
- O: STRK; P: DIP – Azimuth and plunge of the P-axis.
- Q: STRK; R: DIP - Azimuth and plunge of the T-axis.
- S: Q – quality factor of focal solution (2 = best quality; 1 = medium quality; 0 = low quality).
- T: CAT - category of the single focal mechanism (NF = normal fault; NS = normal-strike; SS = strike-slip; TF = thrust fault; TS = thrust-strike; HV = horizontal-vertical).

## Technical Validation

To assess the reliability of the new catalogue we show the distribution of both the vertical and horizontal errors of the 3D hypocentres that were estimated directly from the posterior covariance matrices provided by tomoDDPS for each event. These errors account for the uncertainties in absolute P- and S-wave arrival times, as well as the spatial distribution of stations and the 3D velocity model used. Relative (event-to-event) errors, computed using differential travel-times, are generally lower than absolute errors.

**Fig. 7:**
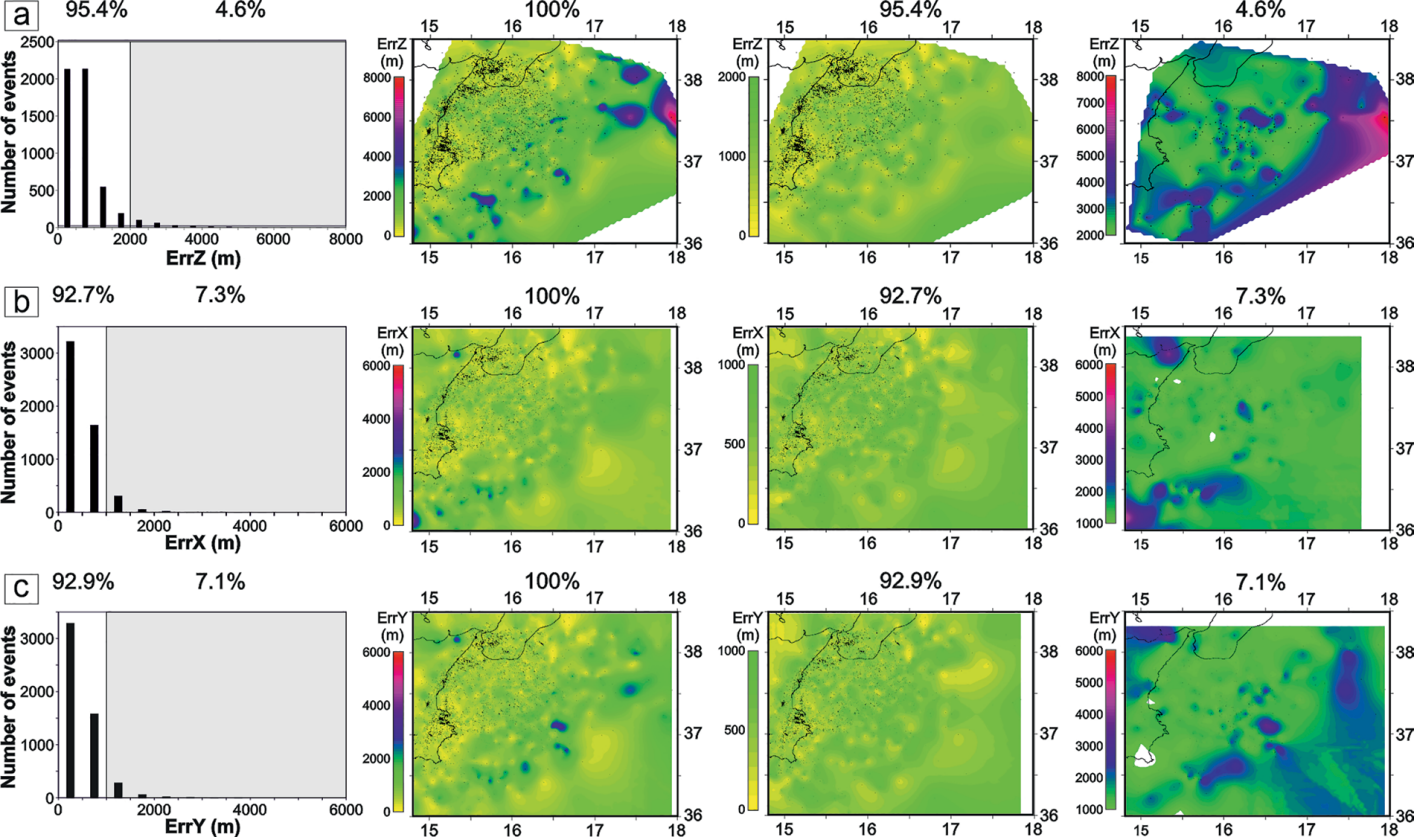
Vertical and horizontal relative location uncertainties of the relocated earthquakes. (**a**) Distribution and spatial pattern of vertical errors (ErrZ). The histogram (left) shows that 95.4% of the events have uncertainties < 2 km, while the maps illustrate the spatial variability of ErrZ across the study region, with higher uncertainties reaching values up to ~8 km. (**b**) Horizontal uncertainties along the E–W direction (ErrX). The histogram indicates that 92.7% of the events have ErrX < 1 km, with maximum values up to 6 km. (**c**) Horizontal uncertainties along the N–S direction (ErrY). As with ErrX, most events (92.9%) exhibit uncertainties < 1 km, and values remain below 6 km throughout the study area. All the maps show that larger uncertainties are concentrated near the margins of the area.

Vertical errors at each location are less than 2 km in about 95.4% of the events (Fig. 7a); only 239 earthquakes (4.6% of the events) exceeded this value, with an upper boundary of less than 8 km (Fig. 7a). These hypocentres affected by higher depth errors are associated with sectors characterized by a lower tomographic model resolution. Considering vertical errors > 5 km (only 8 earthquakes), the distribution of these events lies at the margins of the study area, and at more than 38 km of depth.

Horizontal uncertainties at each location are comparable along the E-W and N-S directions and are generally smaller than the vertical ones. Along the E-W direction (Fig. 7b), about the 92.7% of the events exhibit errors less than 1 km, while 385 earthquakes (7.3%) exceed this value. Similarly, along the N-S direction (Fig. 7c) about the 92.9% of the events have uncertainties below 1 km, with 373 earthquakes (7.1%) above this threshold. In both directions, horizontal uncertainties remain below 6 km. As observed for the depth uncertainties, the largest horizontal errors are distributed in the peripheral areas of the region, depending from the spatial variability in the resolution of the 3D velocity model^7^.

**Fig. 8:**
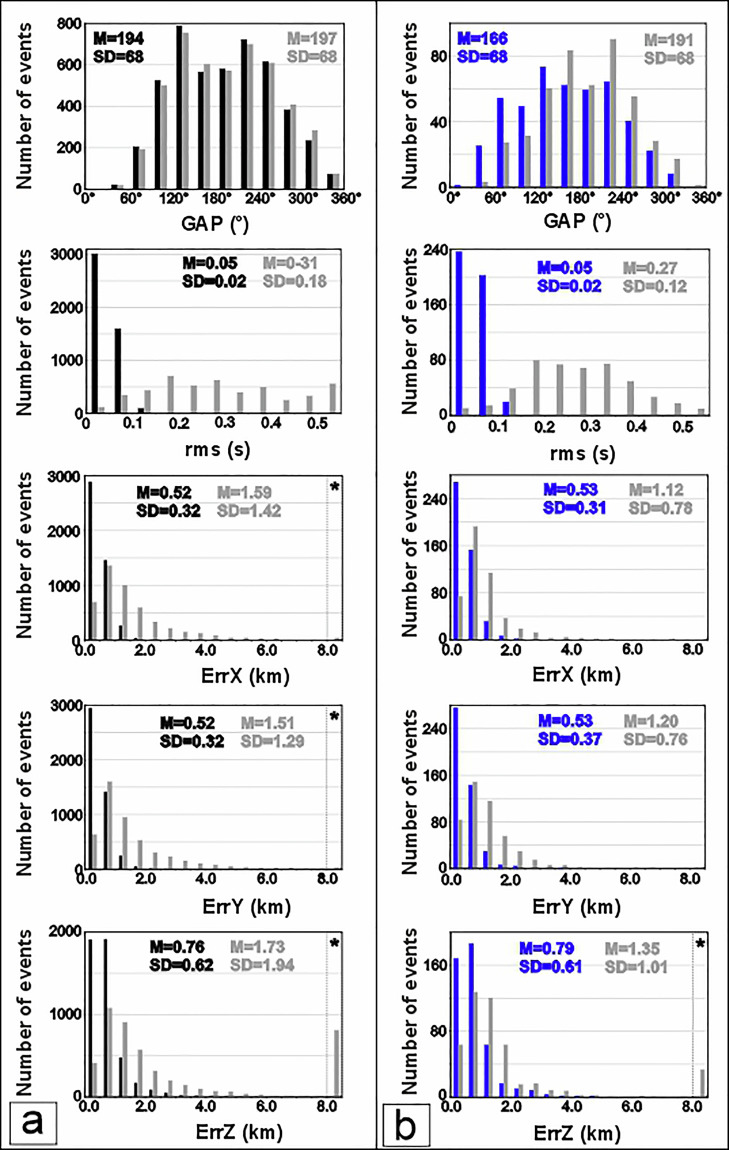
Comparison between relocated earthquakes (black and blue histograms) and original locations from bulletins (grey histograms). (**a**) Distributions of azimuthal GAP, rms travel-time residuals, and horizontal (ErrX, ErrY) and vertical (ErrZ) location uncertainties for the earthquakes relocated in our catalogue (black histograms) compared with the corresponding bulletin solutions (grey histograms). (**b**) Same comparison as in (**a**), but for earthquakes relocated also using available additional data from seafloor stations (blue histograms), compared with the locations of the same earthquakes from bulletins (grey histograms). For each histogram, mean (M) and standard deviation (SD) values are reported. Relocated events (both without and with travel-times data recorded from seafloor stations) show systematically smaller rms values, reduced azimuthal GAP, and lower horizontal and vertical uncertainties, highlighting a clear improvement in earthquake location quality, particularly when offshore station data are included. The asterisk within the grey hatched area indicates values that fall outside the limits of the figure or values that are fixed or not determined in the bulletins.

The quality and reliability of the earthquake locations included in our dataset were assessed by comparing relocated hypocentral parameters with those reported in the original seismic bulletins (Fig. 8). The use of a three-dimensional (3D) velocity model (black histograms in Fig. 8a) and the introduction of seismic data recorded by the OBSs and NEMO-SN1 stations (blue histograms in Fig. 8b), allowed an evaluation of the impact of improved network geometry on location accuracy.

Statistical distributions of azimuthal gap (GAP), root mean square of travel-time residuals (rms), and horizontal (ErrX, ErrY) and vertical (ErrZ) location uncertainties show systematic improvements for the relocated events. In particular, relocations performed with the 3D velocity model produced lower rms values and reduced location uncertainties compared to bulletin solutions, indicating a better fit between observed and calculated travel-times. The inclusion of offshore seismic stations further enhanced this improvement, leading to a significant reduction in azimuthal GAP and a marked decrease in both horizontal and vertical errors.

Overall, the comparison demonstrates that the relocated catalogues provide more accurate and better-constrained earthquake locations than the original bulletin, especially for offshore events. These results (as previously shown by 7, 14, 16, 19) confirm the robustness of the relocation procedure and support the use of this dataset for high-resolution seismotectonic and geodynamic analyses in the western Ionian Sea.

## Data Availability

Original earthquake parameters (locations and travel-times) are available on the INGV institutional websites (https://bsi.ingv.it/it/archivio-dati; http://terremoti.ingv.it/; https://www.ct.ingv.it/index.php/monitoraggio-e-sorveglianza/banche-dati-terremoti/terremoti). All data are shared under the Creative Commons Attribution 4.0 International use license (without restrictions) and available at the links: https://oedatarep.ct.ingv.it/records/8vxp0-wtp69 (URL), 10.13127/wiseloc/1990-2019; and https://oedatarep.ct.ingv.it/records/jn7kb-xf626 (URL), 10.13127/wisefm/1990-2019.

## Code availability

All codes used in this study are described in detail in the Methods section. Surfer was used to plot events and focal mechanisms. Corel Draw 2020 was used for creating all the figures.
